# Stability of Three Different Sanitary Shoes on Healthcare Workers: A Cross-Sectional Study

**DOI:** 10.3390/ijerph16122126

**Published:** 2019-06-16

**Authors:** José Manuel Sánchez-Sáez, Patricia Palomo-López, Ricardo Becerro-de-Bengoa-Vallejo, César Calvo-Lobo, Marta Elena Losa-Iglesias, Andrés López-del-Amo-Lorente, Daniel López-López

**Affiliations:** 1 Research, Health and Podiatry Unit, Department of Health Sciences, Faculty of Nursing and Podiatry, Universidade da Coruña Ferrol, 15403 Ferrol, Spain; jose.m.sanchez.saez@udc.es (J.M.S.-S.); daniel.lopez.lopez@udc.es (D.L.-L.); 2 University Center of Plasencia, University of Extremadura, 10600 Plasencia, Spain; patibiom@unex.es; 3 Faculty of Nursing, Physiotherapy and Podiatry, Complutense University of Madrid, 28040 Madrid, Spain; ribebeva@ucm.es; 4 Nursing and Physical Therapy Department, Faculty of Health Sciences, University of León, Ponferrada, 24401 León, Spain; 5 Faculty of Health Sciences, Rey Juan Carlos University, 28922 Alcorcón, Spain; marta.losa@urjc.es; 6 Faculty of Health Sciences, Catholic University of Murcia, 30107 Murcia, Spain; alopez@ucam.edu

**Keywords:** health personnel, personal protective equipment, postural balance, shoes

## Abstract

Background: The main purpose of this research was to determine the stability of three different sanitary shoes on nurses with eyes open and closed with respect to barefoot condition. In addition, the secondary aim was to determine the reliability of stability measurements under these different conditions. Methods: A crossover quasi-experimental study (NCT03764332) was performed. Twenty-six nurses who wore different sanitary shoes (Eva Plus Ultralight^®^, Gym Step^®^ and Milan-SCL Liso^®^) were evaluated with respect to barefoot condition for stability measures on the Podoprint^®^ podobarometric and stabilometry tool and with eyes open and closed. Furthermore, the reliability of stability measurements was determined by the intraclass correlation coefficient (ICC) under these different conditions. Results: Between-groups comparisons of the static and stabilometry podobarometric data with eyes open showed statistically significant differences (*p* < 0.05). Milan-SCL Liso^®^ sanitary shoes improved podobarometric data of forefoot force and distribution with respect to barefoot condition. Eva Plus Ultralight^®^ and Gym Step^®^ sanitary shoes increased the stroke length mean, stroke surface mean, and anterior speed mean as well as reduced *y* axis displacement mean with respect to barefoot condition. Similar findings were determined for measurements with eyes closed. ICCs ranged from poor to excellent reliability (ICC = 0.010–0.995). Conclusions: Sanitary shoes improved podobarometric and stabilometry stability with respect to barefoot condition.

## 1. Introduction

Nurses stand and walk for extended periods, habitually for a twelve-hour shift walking between eight to ten kilometers [1]. This setup showed psychological and physicall exigencies that may produce musculoskeletal disorders and postural problems [2,3]. Therefore, footwear of nurses may be an important issue for overall health and reduce high rates of musculoskeletal disorders prevalence between 32–90% which are related to: (1) complaints of discomfort, (2) foot and ankle conditions, (3) fractures, (4) joint pain problems, (5) lower back disorders, (6) postural instability, (7) neuropathic injuries, (8) skin infections, and (9) vascular alterations that may affect the healthcare work activities in the daily routine [4,5,6,7].

Further, various studies have researched different types of determinants, including activities of lifestyle routine of the people, work activity, and psychosocial factors related to foot disorders and different anatomical places among nurses. Nevertheless, a small number of studies has focused on the determinants associated with foot and ankle problems [8,9]. Specifically, shoes-related determinants may produce important improvements and prevent foot conditions among nurses, having received low attention in previous literature. Thus, the identification of adequate footwear may be very important to set up preventive strategies to reduce the possible foot alterations in nurses and improve their quality of life and welfare.

Previous studies have not yet evaluated static balance in nurses wearing different sanitary shoes through of the Podoprint^®^ podobarometric and stabilometry platform system. 

Therefore, the main purpose of this research was to determine the stability of three different sanitary shoes on nurses with eyes open and closed with respect to barefoot condition, using the Podoprint^®^ podobarometric and stabilometry platform system. In addition, the secondary aim was to determine the reliability of stability measurements under these different conditions. Therefore, we hypothesized in this investigation that sanitary shoes may increase stability in nurses with respect to barefoot condition.

## 2. Materials and Methods

### 2.1. Design and Sample

A crossover quasi-experimental study was carried out and registered at ClinicalTrials.gov (NCT03764332). A sample of twenty-six nurses (10 females and 16 males), aged between 25 and 35 years, was recruited in a private podiatric medical clinic that supplied assessment of foot and ankle problems in the town of Murcia (Spain) for two months from December 2018 to January 2019. In addition, this research was carried out following the Template for Intervention Description and Replication (TIDieR) guidelines and checklist [10].

Nurses were recruited by a nonrandom consecutive sampling protocol and evaluated by repeated measures. The inclusion standard criteria were as follows: (1) nurses without medical injuries and other illness family history, (2) participants being at least eighteen years old, (3) free from exercise for twenty-four hours to prevent the effects associated with the fatigue, and (4) people who completed the consent form. The exclusion standard criteria were as follows: (1) trauma related to medical history of foot and ankle injuries, (2) vascular alterations, (3) neurological disturbances, (4) musculoskeletal impairment, (5) inability to give a signed consent document, and (6) refusal to go on the protocol of our investigation.

### 2.2. Procedure

The baseline measurement record included general information associated with: (1) medical history of general health, (2) sociodemographic factors (sex, age, and work status), (3) comorbid conditions, (4) sports activities, and (5) current medications.

Then, a senior podiatrist completed a physical exam for each nurse who registered anthropometrics measures such as (1) height, (2) weight, and (3) body mass index. 

Afterwards, each nurse was tested for static stability in barefoot condition and while wearing three different types of sanitary shoes (1-Eva Plus Ultralight^®^, 2-Gym Step^®^ and 3-Milan-SCL Liso^®^) with eyes open. The protocol of testing requirements was randomly selected.

**Figure 1:**
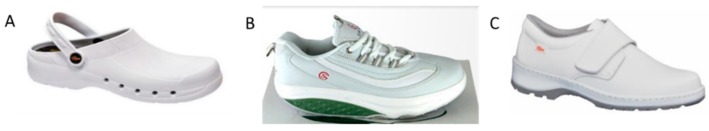
Sanitary shoes: (**A**) Eva Plus Ultralight^®^, (**B**) Gym Step^®^, (**C**) Milan-SCL Liso^®^.

All subjects brought the same three types of footwear to the private podiatric medical clinic. The first model of sanitary shoes (Figure 1A; Eva Plus Ultralight^®^) showed the following characteristics: (1) one anatomical piece of latex free without nonskid, (2) machine-washed to less than forty degrees, (3) foldable rear strap, (4) drilled in the sides to contribute to perspiration, avoid direct penetration of liquids, and facilitate the cleaning, (5) sole with microdots and insole of polyurethane foam with antibacterial treatment and activated carbon, and (6) ultralight at 180 g. 

The second model of sanitary shoes (Figure 1B; Gym Step^®^) showed the following characteristics: (1) air chamber, (2) antislip sole and swinging technology, and (3) anti-injury and padded instep.

The third model of sanitary shoes (Figure 1C; Milan-SCL Liso^®^) showed the following characteristics: (1) footwear with Velcro fastener, (2) machine-washed to less than forty degrees, (3) very breathable microfiber interior, rapid absorption of moisture, and easy drying, (4) anatomically straight structure and rounded shape in the area of the toes, (5) buttress to hold the heel, and (6) cushioned insole of polyurethane foam with shown activated carbon and antibacterial treatment.

Next, this research followed the protocol previously described by Becerro-de-Bengoa et al. [11]. Each healthcare worker stood in a relaxed position on the baropodometric and stabilometry pressure platform Podoprint^®^ (Namrol Group, Barcelona, Spain) and during the simulated walking and gait posture for fifteen seconds. After this period, the participant ceased moving their feet and stood still in a regular manner. With the subject’s feet entirely on the pressure platform and at a comfortable position angle, each participant with eyes open looked straight ahead and kept up the arms close to his/her body. Plantar foot pressure was recorded for both feet, simultaneously measured for thirty seconds. If the participant moved in this period, the findings were discarded, and the test was repeated until it was successfully performed. No practice test was conducted. The static values included in each feet evaluation included various parameters, such as: (1) forefoot (urface (cm²), force (%), distribution (%)), (2) rearfoot (surface (cm²), force (%), distribution (%)) and (3) foot (surface (cm²), force (%), maximum pressure (g/cm^2^), medium pressure (g/cm^2^)). The same test was used for control postural tests with eyes closed when using the Podoprint^®^ baropodometric and stabilometry platform system.

### 2.3. Ethical and Legal Considerations

The investigation was approved by the institutional Research Committee of the Bioethics in the Universidad Rey Juan Carlos, in Móstoles, Spain and was recorded in the ClinicalTrials.gov (NCT03764332). Further, all informants signed the consent form and gave it to the people who wrote it in order for it to be incorporated in our investigation protocol. 

Furthermore, this investigation was performed in accordance with international standards for biomedical experimentation, including the Declaration of Helsinki (version 2008) and other institutions.

### 2.4. Sample size Calculation

The sample size was calculated by the software of the Unit of Clinical Epidemiology and Biostatistics from the University Hospital Complex of A Coruña, Universidade da Coruña (www.fisterra.com), Spain. This sample size calculation was based on a similar prior study which investigated the shoe effects on the stability of a healthy adult population [12], showing general static sway stability values (cm) of 6.13 ± 3.39 cm in barefoot condition and 3.4 ± 2.00 cm while wearing shoes, as well as a two-tailed hypothesis, an α level of 0.05, and a desired power analysis of 80% with a β level of 20%. Thus, a sample size of 16 subjects was estimated.

### 2.5. Statistical Analysis

All data were analyzed using the Statistical Package for Social Sciences (SPSS, v20.0) with a pre-established α level of 0.05, a confidence interval (CI) of 95%, and a *p* value < 0.05 as statistically significant.

Data were analyzed to determine normality distribution using the Shapiro–Wilk test, showing a normal distribution if *p* ≥ 0.05. The descriptive statistical analysis was carried out using the mean ± SD and the 95% CI. 

The intraclass correlation coefficients (ICC) were calculated through a bidirectional randomized effects model, using isolated measurements and absolute concordance in order to determine reliability and test–retest reliability for each parameter [13]. ICC value interpretation was categorized as poor (ICC < 0.40), fair (ICC = 0.40–0.59), good (ICC = 0.60–0.74), and excellent (ICC = 0.75–1.0) [14]. According to Portney and Watkins [15], reliability coefficients with an ICC > 0.90 improve the probability of the available clinical measurements.

In order to compare the conditions, *t*-tests for independent samples or Mann–Whitney U test were used according to normal or no-normal data distribution, respectively. Furthermore, paired *t*-tests or Wilcoxon signed rank tests were used to evaluate systematic between-sessions differences according to normal or no-normal data distribution, respectively.

The interaction effects of the 4 shoe conditions and the 2 eyes conditions during anterior–posterior (AP) and mid-lateral (ML) postural balance were separately analyzed, using a two-way analysis of variance (ANOVA) for parametric data or Kruskal–Wallis for nonparametric data. For the main statistically significant differences of the shoe conditions, post-hoc Tukey analyses were used to determine these differences [16].

## 3. Results

### 3.1. Descriptive Data 

The sample included 26 participants, 16 men and 10 women, whose descriptive data are shown in Table 1.

### 3.2. Reliability of the Static Podobarometric Data with Eyes Open 

The ICCs of the static podobarometric data with eyes open ranged from fair to excellent reliability (ICC = 0.437–0.990) and are shown in Table 2.

### 3.3. Reliability of the Stabilometry Data with Eyes Open 

The ICCs of the stabilometry data with eyes open ranged from poor to excellent reliability (ICC = 0.050–0.962) and are shown in Table 3.

### 3.4. Comparison of the Static Podobarometric Data with Eyes Open between Groups 

The between-groups comparisons of the static podobarometric data with eyes open showed statistically significant differences (*p* < 0.05) and are shown in Table 4. Indeed, Milan-SCL Liso^®^ shoes improved podobarometric data of forefoot force and distribution with respect to barefoot condition. The maximum force peak was improved with various sanitary shoes, being the best improvement produced by the Eva Plus Ultralight^®^ shoes.

### 3.5. Comparison of the Stabilometry Data with Eyes Open between Groups

The comparisons of the stabilometry data with eyes open showed statistically significant differences (*p* < 0.05) and are shown in Table 5. The Milan-SCL Liso^®^ and Gym Step^®^ sanitary shoes increased the stroke length mean, stroke surface mean, and anterior speed mean as well as reduced the *y* axis displacement mean with respect to barefoot condition. In addition, Gym Step^®^ shoes decreased the length/surface mean and increased the lateral and swing speed means with respect to barefoot condition.

### 3.6. Reliability of the Static Podobarometric Data with Eyes Closed

The ICCs of the static podobarometric data with eyes closed ranged from good to excellent reliability (ICC = 0.653–0.995) and are shown in Table 6.

### 3.7. Reliability of the Stabilometry Data with Eyes Closed 

The ICCs of the stabilometry data with eyes closed ranged from poor to excellent reliability (ICC = 0.010–0.954) and are shown in Table 7.

### 3.8. Comparison of the Static Podobarometric Data with Eyes Closed between Groups 

The between-groups comparisons of the static podobarometric data with eyes closed showed statistically significant differences (*p* < 0.05) for wearing different sanitary shoes with respect to barefoot condition and are shown in Table 8. Similar statistically significant differences were determined with respect to measurements with eyes closed (Table 4), showing greater means values in similar outcome measurements.

### 3.9. Comparison of the Stabilometry Data with Eyes Closed Between Groups 

The comparisons of the stabilometry data with eyes closed showed statistically significant differences (*p* < 0.05) and are shown in Table 9. The Milan-SCL Liso^®^ and Gym Step^®^ sanitary shoes decreased the length/surface mean with respect to barefoot condition. In addition, all sanitary shoes decreased the *y* axis displacement mean with respect to barefoot condition. The Gym Step^®^ shoe increased the lateral speed mean, while this measurement was reduced for the other two types of sanitary shoes, with regard to barefoot condition. The Milan-SCL Liso^®^ and Gym Step^®^ sanitary shoes reduced the length/surface mean, while the swing speed mean was increased for Gym Step^®^ shoes and reduced for Milan-SCL Liso^®^ shoes with respect to barefoot condition. Finally, Gym Step^®^ shoes increased the stroke surface and anterior speed means with regard to barefoot condition. Further, similar statistically significant differences were determined with respect to eyes closed measurements (Table 5), showing greater means values in similar outcome measurements.

## 4. Discussion

The main objective of this study was to determine and examine the control postural measurements in barefoot condition and wearing various sanitary shoes, with eyes open and closed, using the Podoprint^®^ baropodometry and stabilometry platform system.

Footwear is essential for nurses in order to improve overall health and reduce high rates of prevalence of musculoskeletal disorders, and inadequate shoes can produce an increased risk for foot disorders in healthcare workers [1,5]. 

Although some investigations have assessed the efficacy of adequate shoe interventions in nurses [17,18], prior studies have not studied the footwear effects on postural control related to different sanitary shoes.

According to our research, Milan-SCL Liso^®^ sanitary shoes improved podobarometric and stabilometric data of forefoot force and distribution with respect to barefoot condition. Milan-SCL Liso^®^ and Gym Step^®^ sanitary shoes too increased the stroke length mean, stroke surface mean, and anterior speed mean as well as reduced *y* axis displacement mean with respect to barefoot condition. Our findings were in accordance with prior findings by Chiu et al., indicating that comfortable footwear was related to a footbed and heel height approximately from 1.8 to 3.6 cm [18].

According to the used measurement protocol described by Becerro de Bengoa Vallejo et al. [11], the platform reliability showed ICCs from 0.566 to 0.877 for static intrasession reliability, ICCs from 0.325 to 0.874 for dynamic intrasession reliability, ICCs from 0.932 to 0.979 for static intersession reliability, and ICCs from 0.822 to 0.974 for dynamic intersession reliability. To the authors’ knowledge, there is a lack of prior similar studies to compare our reliability results under different sanitary shoes with similar conditions. 

There were several limitations to this investigation. Firstly, this investigation ruled out healthcare workers who used custom footwear or insoles. Secondly, a more diverse (subjects from other countries of the world) and larger sample size could improve the power of this investigation. Finally, the Milan-SCL Liso sanitary shoe model showed some low ICC values in the reliability of stabilometry with eyes open and closed. Further studies are necessary in order to clarify the reason of this poor reliability in some stabilometry parameters. Indeed, anatomic differences in male and female feet may affect stability or preference for wearing one type of shoe or another [19,20].

This manuscript highlights the importance of further investigations on the effects of postural control under different footwear types related to foot problems in order to prevent healthcare workers’ health conditions.

## 5. Conclusions

Sanitary shoes improved podobarometric and stabilometry stability with respect to barefoot condition.

## Figures and Tables

**Table 1:** Descriptive data of the study participants.

Descriptive data	Men (n = 16) Mean ± SD (CI 95%)	Women (n = 10) Mean ± SD (CI 95%)	Total (n = 26) Mean ± SD (CI 95%)
Age (years)	30.06 ± 4.15 (28.47–31.66)	29.50 ± 3.50 (28.15–30.85)	29.85 ± 3.85 (28.36–31.33)
Weight (kg)	74.00 ± 14.49 (68.43–79.57)	56.20 ± 4.96 (54.29–58.11)	67.15 ± 14.59 (61.55–72.76)
Height (cm)	1.75 ± 0.05 (1.72–1.77)	1.63 ± 0.03 (1.61–1.64)	1.70 ± 0.07 (1.67–1.73)
BMI (kg/m^2^)	24.30 ± 4.66 (22.51–26.09)	21.32 ± 2.23 (20.46–22.17)	23.15 ± 4.13 (21.57–24.74)

Abbreviations: SD, standard deviation; CI 95%, 95% confidence interval; BMI, body mass index.

**Table 2:** Reliability of the static podobarometric data with eyes open.

Static Podobarometric Data with Eyes Open	Barefoot ICC (95% CI)	Eva Plus Ultralight^®^ Shoes ICC (95% CI)	Milan-SCL Liso^®^ Shoes ICC (95% CI)	Gym Step^®^ ICC (95% CI)
Left forefoot surface (cm²)	0.579 (0.197–0.797)	0.673 (0.381–0.842)	0.616 (0.259–0.816)	0.791 (0.596–0.900)
Left forefoot force (%)	0.752 (0.531–0.880)	0.519 (0.070–0.770)	0.707 (0.439–0.859)	0.839 (0.690–0.923)
Left forefoot distribution (%)	0.762 (0.550–0.885)	0.460 (−0.042–0.741)	0.709 (0.442–0.860)	0.851 (0.713–0.928)
Left rearfoot surface (cm²)	0.940 (0.885–0.971)	0.949 (0.902–0.975)	0.972 (0.946–0.986)	0.960 (0.923–0.981)
Left rearfoot force (%)	0.795 (0.611–0.901)	0.511 (0.060–0.765)	0.730 (0.479–0.871)	0.688 (0.394–0.851)
Left rearfoot distribution (%)	0.744 (0.516–0.876)	0.437 (−0.083–0.730)	0.671 (0.363–0.842)	0.851 (0.715–0.929)
Left foot surface (cm^2^)	0.698 (0.416–0.856)	0.856 (0.726–0.931)	0.848 (0.709–0.927)	0.868 (0.746–0.937)
Left foot force (%)	0.822 (0.661–0.914)	0.813 (0.645–0.910)	0.790 (0.602–0.899)	0.598 (0.241–0.805)
Left foot maximum pressure (g/cm^2^)	0.948 (0.901–0.975)	0.961 (0.925–0.981)	0.941 (0.887–0.972)	0.894 (0.796–0.949)
Left foot medium pressure (g/cm^2^)	0.974 (0.951–0.988)	0.974 (0.949–0.987)	0.956 (0.915–0.979	0.853 (0.719–0.930)
Left foot weight (kg)	0.984 (0.969–0.992)	0.990 (0.981–0.995)	0.989 (0.979–0.995)	0.984 (0.969–0.992)
Right forefoot surface (cm²)	0.647 (0.325–0.830)	0.743 (0.513–0.875)	0.749 (0.516–0.880)	0.772 (0.559–0.891)
Right forefoot force (%)	0.783 (0.583–0.896)	0.294 (−0.321–0.656)	0.767 (0.553–0.888)	0.854 (0.721–0.930)
Right forefoot distribution (%)	0.804 (0.626–0.905)	0.486 (0.027–0.751)	0.768 (0.555–0.888)	0.825 (0.665–0.916)
Right rearfoot surface (cm²)	0.947 (0.898–0.974)	0.942 (0.889–0.972)	0.960 (0.922–0.981)	0.963 (0.929–0.982)
Right rearfoot force (%)	0.785 (0.590–0.896)	0.740 (0.509–0.874)	0.768 (0.557–0.888)	0.814 (0.643–0.911)
Right rearfoot distribution (%)	0.765 (0.556–0.886)	0.657 (0.356–0.833)	0.806 (0.626–0.907)	0.821 (0.658–0.914)
Right foot surface (cm^2^)	0.869 (0.751–0.937)	0.866 (0.745–0.935)	0.885 (0.780–0.945)	0.871 (0.752–0.938)
Right foot force (%)	0.822 (0.661–0.914)	0.813 (0.645–0.910)	0.790 (0.602–0.899)	0.496 (0.028–0.759)
Right foot maximum pressure (g/cm^2^)	0.904 (−0.727–0.570)	0.942 (0.889–0.972)	0.961 (0.926–0.981)	0.927 (0.861–0.965)
Right foot medium pressure (g/cm^2^)	0.974 (0.949–0.987)	0.969 (0.940–0.985)	0.962 (0.927–0.982)	0.925 (0.857–0.964)
Right foot weight (kg)	0.990 (0.982–0.995)	0.993 (0.987–0.997)	0.990 (0.981–0.995)	0.989 (0.980–0.995)

Abbreviations: 95% CI, lower and upper limits of the 95% confidence interval; ICC, intraclass correlation coefficient.

**Table 3:** Reliability of the stabilometry data with eyes open.

Stabilometry Data with Eyes Open	Barefoot ICC (95% CI)	Eva Plus Ultralight^®^ Shoes ICC (95% CI)	Milan-SCL Liso^®^ Shoes ICC (95% CI)	Gym Step^®^ ICC (95% CI)
Stroke length mean (mm)	0.517 (0.067–0.768)	0.256 (−0.427–0.642)	0.576 (0.204–0.794)	0.674 (0.381–0.842)
Stroke Surface mean (mm^2^)	0.309 (−0.349–0.671)	0.309 (−0.313–0.666)	0.050 (−0.868–0.549)	0.734 (0.489–0.872)
Length/surface mean (mm/mm^2^)	0.108 (−0.739–0.575)	0.447 (−0.55–0.733)	0.169 (−0.618–0.604)	0.483 (0.006–0.752)
Swing speed mean (mm/s)	0.496 (0.040–0.757)	0.349 (−0.239–0.686)	0.578 (0.208–0.794)	0.651 (0.339–0.831)
Lateral speed mean (mm/s)	0.430 (−0.073–0.723)	0.246 (−0.470–0.640)	0.608 (0.263–0.809)	0.646 (0.334–0.827)
Anterior speed mean (mm/s)	0.459 (−0.024–0.738)	0.529 (0.117–0.771)	0.507 (0.070–0.760)	0.672 (0.378–0.841)
*x* axis displacement mean (mm)	0.758 (0.539–0.883)	0.721 (0.466–0.866)	0.905 (0.818–0.954)	0.617 (0.273–0.815)
*y* axis displacement mean (mm)	0.926 (0.858–0.964)	0.946 (0.898–0.974)	0.919 (0.845–0.961)	0.962 (0.926–0.982)

Abbreviations: 95% CI, lower and upper limits of the 95% confidence interval; ICC, intraclass correlation coefficient.

**Table 4:** Comparison of the static podobarometric data with eyes open between groups.

Static Podobarometric Data with Eyes Open	Barefoot Mean ± SD (95% CI) n = 26	Eva Plus Ultralight^®^ Shoes Mean ± SD (95% CI) n = 26	Milan-SCL Liso^®^ Shoes Mean ± SD (95% CI) n = 26	Gym Step^®^ Mean ± SD (95% CI) n = 26	*P*-Value Barefoot vs. Eva Plus Ultralight^®^ Shoes	*P*-value Barefoot vs. Milan-SCL Liso^®^ Shoes	*P*-value Barefoot vs. Gym Step^®^
Left forefoot surface (cm²)	37.28 ± 11.86 (32.72–41.84)	53.782 ± 14.17 (48.33–59.23)	41.69 ± 15.61 (35.69–47.69)	26.10 ± 16.89 (19.61–32.60)	0.001 *	0.023 *	0.003 *
Left forefoot force (%)	15.79 ± 4.41 (14.10–17.49)	17.40 ± 2.83 (16.31–18.48)	13.86 ± 4.88 (11.98–15.73)	8.71 ± 6.20 (6.32–11.09)	0.124	0.008 *	0.000 *
Left forefoot distribution (%)	32.81 ± 9.20 (29.27–36.35)	36 ± 5.73 (33.80–38.20)	28.15 ± 9.55 (24.48–31.82)	18.06 ± 13.14 (13.01–23.12)	0.146	0.002 *	0.001 *
Left rearfoot surface (cm²)	62.78 ± 19.93 (55.12–70.44)	68.92 ± 10.00 (65.08–72.77)	74.27 ± 15.85 (68.18–80.36)	83.23 ± 18.76 (76.02–90.44)	0.071	0.002 *	0.001 *
Left rearfoot force (%)	32.33 ± 4.94 (30.43–34.23)	31.10 ± 3.18 (29.88–32.33)	35.26 ± 4.86 (33.39–37.13)	38.92 ± 6.91 (36.27–41.58)	0.451	0.001 *	0.001 *
Left rearfoot distribution (%)	67.06 ± 8.97 (63.62–70.51)	64.03 ± 5.62 (61.87–66.18)	71.54 ± 9.74 (67.79–75.28)	81.99 ± 13.16 (76.93–87.04)	0.142	0.003 *	0.000 *
Left foot surface (cm^2^)	104.37 ± 39.19 (89.31–119.44)	122.44 ± 21.55 (114.15–130.72)	115.92 ± 23.82 (106.77–125.08)	109.24 ± 20.20 (101.48–117.01)	0.001 *	0.006 *	0.154
Left foot force (%)	48.00 ± 2.65 (46.98–49.02)	48.47 ± 2.19 (47.63–49.32)	49.13 ± 2.30 (48.24–50.01)	48.10 ± 1.78 (47.42–48.79)	0.406	0.109	0.951
Left foot maximum pressure (g/cm^2^)	848.67 ± 224.00 (762.57–934.77)	578.69 ± 122.27 (531.69–625.69)	723.46 ± 177.87 (655.09–791.83)	728.92 ± 125.37 (680.73–777.11)	0.001 *	0.003 *	0.003 *
Left foot medium pressure (g/cm^2^)	360.00 ± 99.38 (321.80–398.20)	290.08 ± 61.35 (266.49–313.66)	315.04 ± 76.46 (285.65–344.43)	344.03 ± 64.74 (319.14–368.91)	0.001 *	0.001 *	0.313
Left foot weight (kg)	32.46 ± 6.37 (30.01–34.91)	32.72 ± 6.57 (30.19–35.24)	33.27 ± 7.09 (30.54–36.00)	32.68 ± 6.33 (30.25–35.11)	0.410	0.146	0.784
Right forefoot surface (cm²)	37.71 ± 13.05 (32.69–42.72)	55.36 ± 16.21 (49.13–61.59)	42.74 ± 17.22 (36.12–49.36)	25.54 ± 16.65 (19.14–31.94)	0.001 *	0.089	0.001 *
Right forefoot force (%)	17.36 ± 5.21 (15.36–19.36)	19.78 ± 3.68 (18.37–21.20)	15.35 ± 5.07 (13.40–17.30)	9.50 ± 7.13 (6.76–12.24)	0.049 *	0.022 *	0.001 *
Right forefoot distribution (%)	33.26 ± 10.10 (29.38–37.14)	37.37 ± 6.07 (35.04)	30.23 ± 10.15 (26.33–34.13)	18.59 ± 13.63 (13.35–28.83)	0.080	0.054	0.001 *
Right rearfoot surface (cm²)	68.27 ± 18.59 (61.12–75.42)	69.14 ± 9.83 (65.36–72.92)	72.83 ± 15.13 (67.02–78.65)	86.21 ± 17.84 (79.35–93.06)	0.501	0.029 *	0.001 *
Right rearfoot force (%)	34.88 ± 5.44 (32.80–36.97)	32.06 ± 3.97 (30.54–33.59)	35.37 ± 5.46 (33.27–37.47)	42.18 ± 7.06 (39.47–44.89)	0.060	0.581	0.001 *
Right rearfoot distribution (%)	65.94 ± 10.96 (61.72–70.15)	62.18 ± 6.78 (59.57–64.79)	69.79 ± 9.87 (66.00–73.59)	81.51 ± 13.73 (76.23–86.79)	0.109	0.022 *	0.001 *
Right foot surface (cm^2^)	106.45 ± 26.73 (96.18–116.72)	124.76 ± 23.89 (115.58–133.94)	115.01 ± 26.32 (104.90–125.13)	111.44 ± 19.28 (104.02–118.85)	0.001 *	0.004 *	0.258
Right foot force (%)	52.00 ± 2.65 (50.98–53.02)	51.53 ± 2.19 (50.68–52.37)	50.87 ± 2.30 (49.99–51.76)	51.79 ± 1.89 (51.07–52.52)	0.406	0.109	0.855
Right foot maximum pressure (g/cm^2^)	940.94 ± 642.72 (693.89–1187.99)	620.65 ± 142.52 (565.87–675.44)	753.29 ± 209.11 (672.92–833.67)	770.04 ± 171.70 (704.04–836.04)	0.001 *	0.004 *	0.063
Right foot medium pressure (g/cm^2^)	366.65 ± 95.16 (330.08–403.23)	305.90 ± 70.61 (278.76–333.04)	328.08 ± 80.34 (297.20–358.96)	356.45 ± 59.89 (333.43–379.47)	0.001 *	0.001 *	0.778
Right foot weight (kg)	34.41 ± 8.08 (31.31–37.52)	34.76 ± 8.28 (31.58–37.94)	33.37 ± 7.64 (30.44–36.31)	35.36 ± 7.84 (32.34–38.37)	0.114	0.024 *	0.925

Abbreviations: 95% CI, lower and upper limits of the 95% confidence interval; SD, standard deviation. * Statistical significance was set at *p* < 0.05 with a 95% confidence interval.

**Table 5:** Comparison of the stabilometry data with eyes open between groups.

Stabilometry Data with Eyes Open	Barefoot Mean ± SD (95% CI) n = 26	Eva Plus Ultralight^®^ Shoes Mean ± SD (95% CI) n = 26	Milan-SCL Liso^®^ Shoes Mean ± SD (95% CI) n = 26	Gym Step^®^ Mean ± SD (95% CI) n = 26	*P*-Value Barefoot vs. Eva Plus Ultralight^®^ Shoes	*P*-Value Barefoot vs. Milan-SCL Liso^®^ Shoes	*P*-Value Barefoot vs. Gym Step^®^
Stroke length mean (mm)	87.01 ± 44.54 (69.89–104.13)	100.37 ± 47.04 (82.29–118.45)	97.63 ± 45.78 (80.04–115.23)	163.85 ± 76.47 (134.45–193.24)	0.052	0.008 *	0.001 *
Stroke Surface mean (mm^2^)	82.76 ± 83.81 (50.54–114.98)	118.38 ± 133.40 (67.10–169.66)	109.51 ± 107.56 (68.16–150.85)	185.51 ± 124.50 (137.65–233.37)	0.238	0.032 *	0.001 *
Length/surface mean (mm/mm^2^)	2.39 ± 1.53 (1.81–2.98)	1.63 ± 0.72 (1.35–1.91)	1.94 ± 0.93 (1.59–2.30)	1.11 ± 0.38 (0.96–1.25)	0.017 *	0.178	0.001 *
Swing speed mean (mm/s)	2.11 ± 1.16 (1.66–2.55)	2.39 ± 1.16 (1.95–2.84)	2.25 ± 1.08 (1.83–2.66)	5.04 ± 2.41 (4.11–5.97)	0.104	0.071	0.001 *
Lateral speed mean (mm/s)	1.94 ± 0.75 (1.66–2.23)	1.98 ± 0.83 (1.66–2.30)	2.00 ± 0.74 (1.72–2.28)	3.44 ± 1.50 (2.86–4.02)	0.737	0.309	0.001 *
Anterior speed mean (mm/s)	1.87 ± 1.36 (1.34–2.39)	2.36 ± 1.54 (1.77–2.95)	2.18 ± 1.27 (1.69–2.67)	3.56 ± 2.01 (2.79–4.34)	0.056	0.006 *	0.001 *
*x* axis displacement mean (mm)	4.24 ± 5.44 (2.14–6.33)	2.95 ± 4.32 (1.29–4.61)	2.53 ± 6.56 (0.00–5.05)	4.12 ± 4.88 (2.24–5.99)	0.073	0.131	0.989
*y* axis displacement mean (mm)	−54.02 ± 14.87 (−59.74–−48.31)	−46.07 ± 9.59 (−49.75–−42.38)	−48.82 ± 15.09 (−54.61–−43.02)	−43.23 ± 23.15 (−52.13–−34.33)	0.049 *	0.023 *	0.019 *

Abbreviations: 95% CI, lower and upper limits of the 95% confidence interval; SD, standard deviation. *Statistical significance was set at *p* < 0.05 with a 95% confidence interval.

**Table 6:** Reliability of the static podobarometric data with eyes closed.

Static Podobarometric Data with Eyes Closed	Barefoot ICC (95% CI)	Eva Plus Ultralight^®^ Shoes ICC (95% CI)	Milan-SCL Liso^®^ Shoes ICC (95% CI)	Gym Step^®^ ICC (95% CI)
Left forefoot surface (cm²)	0.935 (0.873–0.969)	0.915. (0.837–0.959)	0.900 (0.808–0.952)	0.895 (0.799–0.949)
Left forefoot force (%)	0.843 (0.700–0.925)	0.653 (0.326–0.834)	0.857 (0.725–0.932)	0.901 (0.811–0.952)
Left forefoot distribution (%)	0.875 (0.760–0.940)	0.688 (0.398–0.850)	0.856 (0.723–0.931)	0.892 (0.793–0.948)
Left rearfoot surface (cm²)	0.944 (0.894–0.973	0.940 (0.886–0.971)	0.961 (0.925–0.981)	0.968 (0.939–0.985)
Left rearfoot force (%)	0.852 (0.717–0.929)	0.815 (0.648–0.910)	0.834 (0.681–0.920)	0.909 (0.826–0.956)
Left rearfoot distribution (%)	0.875 (0.760–0.940)	0.668 (0.357–0.841)	0.850 (0.711–0.928)	0.902 (0.812–0.953)
Left foot surface (cm^2^)	0.935 (0.873–0.969)	0.915 (0.837–0.959)	0.900 (0.808–0.952)	0.895 (0.799–0.949)
Left foot force (%)	0.756 (0.530–0.883)	0.887 (0.781–0.946)	0.818 (0.650–0.912)	0.814 (0.646–0.910)
Left foot maximum pressure (g/cm^2^)	0.995 (0.915–0.979)	0.927 (0.859–0.965)	0.971 (0.944–0.986)	0.932 (0.869–0.967)
Left foot medium pressure (g/cm^2^)	0.976 (0.952–0.989)	0.973 (0.947–0.987)	0.990 (0.982–0.995)	0.935 (0.877–0.969)
Left foot weight (kg)	0.987 (0.974–0.994)	0.994 (0.988–0.997)	0.994 (0.989–0.997)	0.974 (0.950–0.987)
Right forefoot surface (cm²)	0.881 (0.773–0.943)	0.805 (.628–0.906)	0.877 (0.763–0.941)	0.792 (0.603–0.900)
Right forefoot force (%)	0.869 (0.750–0.937)	0.627 (0.287–0.821)	0.900 (0.809–0.952)	0.893 (0.796–0.949)
Right forefoot distribution (%)	0.866 (0.744–0.935)	0.665 (0.336–0.835)	0.897 (0.804–0.950)	0.883 (0.775–0.944)
Right rearfoot surface (cm²)	0.946 (0.897–0.974)	0.961 (0.926–0.981)	0.946 (0.896–0.974)	0.969 (0.941–0.989)
Right rearfoot force (%)	0.876 (0.763–0.940)	0.603 (0.231–0.810)	0.905 (0.819–0.954)	0.852 (0.716–0.929)
Right rearfoot distribution (%)	0.843 (0.701–0.924)	0.657 (0.341–0.835)	0.895 (0.800–0.950)	0.896 (0.801–0.950)
Right foot surface (cm^2^)	0.948 (0.900–0.975)	0.927 (0.860–0.965)	0.925 (0.857–0.964)	0.867 (0.746–0.936)
Right foot force (%)	0.759 (0.535–0.884)	0.890 (0.786–0.947)	0.811 (0.639–0.909)	0.814 (0.646–0.910)
Right foot maximum pressure (g/cm^2^)	0.955 (0.914–0.978)	0.970 (0.943–0.985)	0.984 (0.970–0.993)	0.949 (0.901–0.975)
Right foot medium pressure (g/cm^2^)	0.975 (0.953–0.988)	0.978 (0.958–0.989)	0.992 (0.985–0.996)	0.929 (0.865–0.966)
Right foot weight (kg)	0.991 (0.983–0.996)	0.996 (0.991–0.998)	0.994 (0.988–0.997)	0.992 (0.984–0.996)

Abbreviations: 95% CI, lower and upper limits of the 95% confidence interval; ICC, intraclass correlation coefficient.

**Table 7:** Reliability of the stabilometry data with eyes closed.

Stabilometry Data with Eyes Closed	Barefoot ICC (95% CI)	Eva Plus Ultralight^®^ Shoes ICC (95% CI)	Milan-SCL Liso^®^ Shoes ICC (95% CI)	Gym Step^®^ ICC (95% CI)
Stroke length mean (mm)	0.127 (−0.604–0.569)	0.885 (0.780–0.944)	0.629 (0.307–0.819)	0.938 (0.882–0.970)
Stroke Surface mean (mm^2^)	0.544 (0.143–0.778)	0.844 (0.703–0.925)	0.110 (−0.743–0.577)	0.941 (0.887–0.972=
Length/surface mean (mm/mm^2^)	0.010 (−0.801–0.509)	0.566 (0.161–0.792)	0.353 (−0.254–0.691)	0.428 (−0.097–0.725)
Swing speed mean (mm/s)	0.125 (−0.605–0.568)	0.906 (0.820–0.955)	0.636 (0.319–0.822)	0.936 (0.878–0.969)
Lateral speed mean (mm/s)	0.155 (−0.542–0.582)	0.901 (0.811–0.953)	0.716 (0.466–0.862)	0.885 (0.780–0.945)
Anterior speed mean (mm/s)	0.074 (−0.703–0.544)	0.867 (0.746–0.936)	0.583 (0.220–0.796)	0.945 (0.895–0.973)
*x* axis displacement mean (mm)	0.865 (0.742–0.935)	0.921 (0.849–0.962)	0.862 (0.735–0.934)	0.704 (0.438–0.857)
*y* axis displacement mean (mm)	0.829 (0.672–0.917)	0.851 (0.714–0.928)	0.927 (0.861–0.965)	0.954 (0.913–0.978)

Abbreviations: 95% CI, lower and upper limits of the 95% confidence interval; ICC, intraclass correlation coefficient.

**Table 8:** Comparison of the static podobarometric data with eyes closed between groups.

Static Podobarometric Data with Eyes Closed	Barefoot Mean ± SD (95% CI) n = 26	Eva Plus Ultralight^®^ Shoes Mean ± SD (95% CI) n = 26	Milan-SCL Liso^®^ Shoes Mean ± SD (95% CI) n = 26	Gym Step^®^ Mean ± SD (95% CI) n = 26	*P*-Value Barefoot vs. Eva Plus Ultralight^®^ Shoes	*P*-Value Barefoot vs. Milan-SCL Liso^®^ Shoes	*P*-Value Barefoot vs. Gym Step^®^
Left forefoot surface (cm²)	37.22 ± 12.17 (32.54–41.90)	53.00 ± 13.30 (47.89–58.11)	43.08 ± 17.16 (36.48–49.67)	31.19 ± 18.20 (24.20–38.19)	0.001 *	0.006 *	0.135
Left forefoot force (%)	15.67 ± 4.26 (14.03–17.30)	17.94 ± 2.60 (12.16–15.91)	14.04 ± 4.88 (12.16–15.91)	9.17 ± 5.62 (7.01–11.33)	0.013 *	0.042 *	0.001 *
Left forefoot distribution (%)	32.42 ± 9.15 (28.91–35.94)	36.95 ± 5.66 (34.77–39.12)	28.77 ± 9.67 (25.05–32.49)	18.85 ± 11.50 (14.43–23.26)	0.021 *	0.025 *	0.001 *
Left rearfoot surface (cm²)	65.21 ± 18.68 (58.02–72.39)	68.35 ± 11.13 (64.07–72.63)	76.63 ± 15.22 (70.78–82.48)	92.41 ± 13.96 (87.04–97.78)	0.298	0.001 *	0.001 *
Left rearfoot force (%)	32.38 ± 4.81 (30.54–34.23)	30.81 ± 3.82 (29.34–32.28)	34.29 ± 4.29 (32.65–35.94)	39.51 ± 5.64 (37.35–41.68)	0.128	0.022 *	0.001 *
Left rearfoot distribution (%)	67.63 ± 9.30 (64.05–71.20)	62.94 ± 5.49 (60.82–65.05)	71.17 ± 9.49 (67.52–74.81)	81.04 ± 11.36 (76.67–85.40)	0.021 *	0.025 *	0.001 *
Left foot surface (cm^2^)	103.53 ± 24.78 (93.00–112.05)	121.60 ± 21.82 (113.22–129.99)	119.64 ± 24.27 (110.31–128.97)	123.65 ± 21.91 (115.23–132.08)	0.001 *	0.001 *	0.001 *
Left foot force (%)	47.79 ± 2.23 (46.94–48.65)	48.60 ± 2.25 (47.74–49.47)	48.40 ± 1.99 (47.63–49.16)	48.68 ± 1.74 (48.01–49.35)	0.170	0.128	0.235
Left foot maximum pressure (g/cm^2^)	839.72 ± 180.94 (770.17–909.27)	571.06 ± 120.69 (524.67–617.46)	718.77 ± 183.06 (648.40–789.14)	768.92 ± 165.73 (705.22–832.63)	0.001 *	0.001 *	0.048 *
Left foot medium pressure (g/cm^2^)	346.82 ± 86.55 (313.55–380.09)	288.87 ± 64.36 (264.13–313.61)	303.83 ± 67.74 (277.79–329.87)	339.64 ± 60.44 (316.41–362.87)	0.001 *	0.001 *	0.778
Left foot weight (kg)	32.42 ± 6.56 (29.90–34.94)	32.85 ± 6.54 (30.33–35.36)	32.85 ± 7.16 (30.09–35.60)	33.17 ± 7.09 (30.44–35.89)	0.379	0.170	0.201
Right forefoot surface (cm²)	36.81 ± 12.58 (31.97–41.64)	54.19 ± 15.16 (48.36–60–02)	45.22 ± 18.01 (38.29–52.14)	29.64 ± 19.24 (22.24–37.04)	0.001 *	0.002 *	0.045 *
Right forefoot force (%)	16.77 ± 5.40 (14.69–18.85)	19.76 ± 3.17 (18.54–20.98)	15.87 ± 5.66 (13.70–18.05)	10.08 ± 6.64 (7.53–12.63)	0.003 *	0.231	0.001 *
Right forefoot distribution (%)	32.18 ± 10.22 (28.25–36.11)	38.40 ± 6.27 (35.99–40.81)	30.88 ± 11.01 (26.65–35.11)	19.29 ± 12.45 (14.51–24.08)	0.002 *	0.388	0.001 *
Right rearfoot surface (cm²)	71.65 ± 18.40 (64.58–78.73)	69.79 ± 12.18 (70.29–82.15)	76.22 ± 15.42 (70.29–82.15)	95.24 ± 15.53 (89.27–101.21)	0.861	0.063	0.001 *
Right rearfoot force (%)	35.41 ± 5.85 (33.16–37.66)	31.72 ± 3.42 (30.40–33.03)	35.81 ± 6.29 (33.39–38.23)	41.40 ± 6.39 (38.94–43.85)	0.007 *	0.786	0.001 *
Right rearfoot distribution (%)	67.55 ± 9.64 (63.85–71.26)	61.71 ± 6.73 (59.12–64.29)	69.08 ± 10.92 (64.88–73.27)	80.45 ± 12.38 (75.69–85.21)	0.002 *	0.374	0.001 *
Right foot surface (cm^2^)	108.26 ± 26.05 (98.24–118.27)	124.46 ± 24.41 (115.08–133.85)	121.32 ± 25.24 (111.62–131.02)	124.85 ± 23.41 (115.85–133.84)	0.001 *	0.001 *	0.003 *
Right foot force (%)	52.18 ± 2.24 (51.32–53.04)	51.40 ± 2.24 (50.54–52.26)	51.55 ± 2.01 (50.78–52.33)	51.32 ± 1.77 (50.64–52.00)	0.198	0.128	0.258
Right foot maximum pressure (g/cm^2^)	824.73 ± 160.00 (763.23–886.23)	619.82 ± 154.77 (560.33–679.31)	758.78 ± 213.68 (676.65–840.92)	769.19 ± 195.24 (694.14–844.24)	0.001 *	0.008 *	0.096
Right foot medium pressure (g/cm^2^)	358.21 ± 88.87 (324.05–392.37)	302.26 ± 70.26 (275.25–329.26)	321.94 ± 77.33 (292.21–351.66)	351.65 ± 63.74 (327.15–376.16)	0.001 *	0.001 *	0.509
Right foot weight (kg)	35.38 ± 7.99 (32.31–38.45)	34.99 ± 8.03 (31.90–38.07)	34.92 ± 7.33 (32.11–37.74)	34.88 ± 7.28 (32.09–37.68)	0.449	0.144	0.512

Abbreviations: 95% CI, lower and upper limits of the 95% confidence interval; SD, standard deviation. * Statistical significance was set at *p* < 0.05 with a 95% confidence interval.

**Table 9:** Comparison of the stabilometry data with eyes closed between groups.

Stabilometry Data with Eyes Closed	Barefoot Mean ± SD (95% CI) n = 26	Eva Plus Ultralight^®^ Shoes Mean ± SD (95% CI) n = 26	Milan-SCL Liso^®^ Shoes Mean ± SD (95% CI) n = 26	Gym Step^®^ Mean ± SD (95% CI) n = 26	*P*-Value Barefoot vs. Eva Plus Ultralight^®^ Shoes	*P*-Value Barefoot vs. Milan-SCL Liso^®^ Shoes	*P*-Value Barefoot vs. Gym Step^®^
Stroke length mean (mm)	151.16 ± 86.96 (117.74–184.59)	113.54 ± 53.54 (92.96–134.12)	126.51 ± 52.19 (106.45–146.57)	350.56 ± 66.98 (334.81–386.30)	0.006 *	0.316	0.001 *
Stroke Surface mean (mm^2^)	137.56 ± 119.18 (91.75–183.37)	176.22 ± 259.11 (76.62–275.82)	169.38 ± 174.01 (102.49–236.27)	837.22 ± 200.34 (760.21–914.22)	0.166	0.395	0.001 *
Length/surface mean (mm/mm^2^)	2.12 ± 1.64 (1.49–2.75)	1.53 ± 0.82 (1.22–1.85)	1.49 ± 0.74 (1.20–1.77)	0.88 ± 0.65 (0.63–1.13)	0.137	0.016 *	0.001 *
Swing speed mean (mm/s)	4.73 ± 2.83 (3.64–5.82)	3.46 ± 1.70 (2.81–4.12)	3.91 ± 1.62 (3.29–4.54)	11.18 ± 2.05 (10.39–11.97)	0.005	0.278	0.001 *
Lateral speed mean (mm/s)	3.14 ± 1.67 (2.50–3.79)	2.21 ± 0.91 (1.86–2.56)	2.45 ± 0.97 (2.08–2.82)	6.37 ± 1.22 (5.90–6.84)	0.001 *	0.025 *	0.001 *
Anterior speed mean (mm/s)	3.42 ± 2.30 (2.53–4.30)	2.63 ± 1.42 (2.08–3.17)	3.00 ± 1.37 (2.48–3.53)	9.04 ± 1.70 (8.39–9.70)	0.243	0.889	0.001 *
*x* axis displacement mean (mm)	3.11 ± 6.13 (0.75–5.46)	3.38 ± 5.39 (1.31–5.45)	4.41 ± 5.62 (2.25–6.57)	2.24 ± 5.28 (0.21–4.27)	0.732	0.990	0.657
*y* axis displacement mean (mm)	−53.16 ± 18.08 (−60.11–−46.21)	−44.83 ± 16.70 (−51.25–−38.41)	−45.65 ± 16.96 (−51.01–−40.28)	−43.58 ± 15.44 (−49.51–−37.64)	0.005 *	0.030 *	0.043 *

Abbreviations: 95% CI, lower and upper limits of the 95% confidence interval; SD, standard deviation. * Statistical significance was set at *p* < 0.05 with a 95% confidence interval.
